# Supplementary material for: Are carbon emissions trading and green financial instruments synergistic? -Comprehensive quantitative research based on content analysis
Source: PLoS One. 2024 Mar 7;19(3):e0298601. doi: 10.1371/journal.pone.0298601 (PMC10919601; doi:10.1371/journal.pone.0298601)
Supplement: S1 Data — (ZIP) [file pone.0298601.s001.zip › Data/地级市Z_F工作报告.zip!/╜¡╦╒/╬▐╬2╩╨/└┤╫╘╣1⁄2╓┌║┼í╛┬φ┐╦╩2╛▌═°í┐_2009.docx]

各位代表：

　　现在，我代表无锡市人民政府，向大会报告工作，请予审议，并请市政协委员和其他列席人员提出意见。

2008年工作回顾

　　刚刚过去的2008年，是极不平凡的一年。在中共无锡市委的领导下，市政府以科学发展观为统领，紧紧依靠全市人民，积极应对多重考验，胜利完成了市十四届人大一次会议确定的目标任务。预计，实现地区生产总值4400亿元以上，同比增长13%左右；财政总收入909.2亿元，增长28.6%，其中一般预算收入365.4亿元，同口径增长21.6%；城市居民人均可支配收入、农民人均纯收入分别增长13%和11.5%；全市金融机构各项本外币存贷款余额分别为5483.9亿元和3842.9亿元；城镇登记失业率3.12%；居民消费价格涨幅5.1%；万元地区生产总值能耗下降4.5%，主要污染物化学需氧量和二氧化硫排放总量在2005年基础上均累计削减12%以上，各项工作取得了新的进步。

　　（一）加快结构调整，国民经济保持平稳增长

　　增长动力更趋合理。预计，全社会固定资产投资完成1877亿元，增长12.1%。市场体系日趋完善，消费需求较为旺盛，新增外资大型商场10家，完成农贸市场商场化改造12家。预计，完成社会消费品零售总额1391.5亿元，增长22.6%；商品市场成交额2420亿元。对外贸易继续扩大，预计，实现进出口总额560.4亿美元，增长9.5%；其中出口总额357.9亿美元，增长22%，高新技术产品出口份额提高到47%。经济结构继续优化。积极提升制造业层次，扩大服务业规模，提高农业现代化水平。按国家新标准认定高新技术企业246家，高新技术产业增加值占规模以上工业增加值比重提高到41.5%。软件及服务外包、休闲旅游、动漫创意、现代传媒等产业成为新增长点，预计服务业增加值占地区生产总值的41%。建成规模以上现代农业园区118个，锡山现代农业示范区基本建成。产业竞争力明显增强。大规模集成电路产业制造技术和能力达到国内城市第一。“质量与知识产权立市”战略全面实施，建成全国首个省级卓越绩效管理孵化基地，一汽锡柴荣获首个“市长质量奖”，无锡拥有的“全国标准化专业技术委员会”数量列国内城市之首，专利授权量超过5000件，新增中国驰名商标19件。无锡高新区成为全国首批海外高层次人才创新创业基地之一。预计全社会研发费用占地区生产总值比重达2.1%。

　　（二）深化改革开放，发展动力进一步增强

　　利用外资水平稳步提升。加速壮大优势产业集群，大力建设国家服务外包示范区，全力推进“123”计划，利用外资规模和质量继续攀升。预计，完成到位注册外资31.6亿美元，增长14%，其中高新技术产业和服务业利用外资占比分别提高到50%和22%；引进独立研发中心13家。经济改革纵深推进。优化国有经济布局，推进国有资本向高新技术、地方金融、基础设施和公共服务领域集聚，国有经济整体实力增强。民营经济日益壮大，预计从业人员总数超过165万人，增加值占地区生产总值比重提高到60.7%。加快土地流转创新试验，开展富民合作，组建村级集体经济股份合作社380家、土地股份合作社303家、专业合作经济组织361家。新韩银行、民生银行等4家境内外银行落户无锡，农村小额贷款公司正式运营。行政改革力度加大。取消和停止征收行政事业性收费和政府性基金101项，减轻社会负担5亿元左右。扩大参与式预算改革实施范围，扩大政府采购范围与规模。推动城市管理重心下移，基本实现区级城管机构、人员、经费三到位。深化社会事业改革，进一步完善体制机制。市区市政、环卫养护作业全面实行市场化运作。无锡被评为“中国改革优秀城市”，江阴被确定为全国改革开放三十年18个典型地区之一。

　　（三）统筹城乡发展，城市现代化加速推进

　　城乡空间布局更为优化。实施城乡一体化规划，深化城镇空间体系、区域路网、功能性载体、教文卫体设施布局等规划编制。太湖新城建设全面展开，太湖国际科技园、科教产业园等重点园区初显雏形，老城区重点片区改造加快推进。基础设施功能继续完善。全面启动第三轮“城市建设三年行动纲要”建设任务，积极提升中心城市综合功能。继续加快市区重点道桥建设，全长29.3公里的快速内环全线贯通，凤翔北路、惠钱路、中南路等新改建道路竣工通车，市区新增城市道路73公里。城市快速轨道交通近期建设规划获批，京沪高速铁路和沪宁城际铁路无锡段全面开工建设。229省道江阴段等85公里国省干线完成高标准改造，沪宁高速公路无锡机场互通、锡苏高速公路无锡段相继建成，苏南运河航道升级改造工程全面开工。预计，全市港口完成货物吞吐量1.5亿吨，集装箱运量52万标箱，江阴港被列为大陆首批两岸直航港口之一。无锡机场被批准为国家一类口岸并正式对外开放，全年进出港旅客超过164万人次，货邮吞吐量近4万吨。加强电网建设与改造，建成35千伏及以上变电容量519万千伏安。总投资30亿元的市区长江引水工程竣工投用，双水源供水格局基本形成，自来水水质提前达到国家生活饮用水新标准。农村五件实事全面完成。新建农村道路131公里，修缮农村危桥265座；建成村级“为农服务社”171个，其中农村社区服务中心54家；建成村（居）委健身点620个，6个乡镇文化站实现达标建设；完成村庄环境整治365个，15个镇成为现代化新农村示范镇，农村面貌显著改善。

　　（四）坚持环保优先，生态环境稳定趋好

　　太湖水污染防治强力推进。围绕“十一五”太湖治理目标，落实控源截污、蓝藻打捞、调水清淤、生态修复等各项措施，太湖无锡水域水质得到改善。23条入湖河道的170个排污口全部封堵。市区4568家单位全面截污，全市开发开放园区所有工业和生活污水全部接管处理，市区新增污水管网1362.4公里。列入省目标考核的54家污水处理厂提标改造工作基本完成，市区城镇生活污水处理率达到85%以上。贡湖水源地完成清淤111万立方米，县乡河道疏浚清淤993万立方米，村庄河塘疏浚清淤1310万立方米，打捞蓝藻50万吨。节能减排成效明显。严格落实重点地区、重点行业、重点企业能耗管理和污染治理措施，严格落实工程减排、结构减排、监管减排措施，累计关停“五小”、“三高两低”企业1421家，关停并转沿湖企业53家。加大执法力度，共有80家违法企业作出公开道歉和承诺。无锡成为国家节水型城市。生态建设稳步开展。预计，全市植树造林6900公顷，森林覆盖率提高到22.3%；市区新增城市绿地712万平方米，建成区绿化覆盖率达43%，全长38公里蠡湖环湖绿带全面建成。完成土地复垦整理5546公顷，新增耕地1264公顷，完成矿山坡面复绿40万平方米。城市环境空气质量良好以上天数达343天。预计环境质量综合指数达到83.6。

　　（五）发展社会事业，城乡文明程度明显提升

　　各类教育统筹发展。落实义务教育免收学杂费政策，各级财政共投入9737万元，免费发放教科书1476万余册，惠及学生48万多人。实施中等职业学校国家助学金制度，市级发放助学金9055万元，惠及职业学校学生近6万人。全市26所“公有民办”学校全部依法依规改制到位。105所村办幼儿园完成达标改造，各级财政投入资金5155万元。藕塘职教园区启动区基本建成，6所院校入驻。北大软件与微电子学院无锡产学研合作教育基地投用。卫生事业不断进步。优化医疗卫生资源配置，完善社区卫生服务体系，市级财政安排专项资金1亿元，全面完成了城区23个社区卫生服务中心规范化建设。开展社区卫生惠民服务，减免社区就诊费用2684.4万元。资助6.8万名农村困难人员免费参加新型农村合作医疗，农村居民参保率提高到99.8%，人均筹资256元。市残疾人康复中心正式投用。健康城市创建全面启动，新增国家卫生镇9个、省卫生镇3个。文化名城活力焕发。阖闾城遗址保护性考古发掘圆满完成，惠山古街核心区一期完成修复，清名桥街区一期主体工程基本竣工。无锡博物院、鸿山遗址博物馆、灵山胜境三期、何振梁与奥林匹克陈列馆等建成开放。宜兴成为省级历史文化名城。群众性文化体育活动精彩纷呈，无锡籍运动员参加北京残奥会获金牌2枚。扩大对外交往，新增国际友城6个。建立健全人口管理服务体系，稳定优生优育水平，提高人口素质。对持独生子女父母光荣证退休的企业人员兑现奖励政策，发放一次性奖励金2.7亿元。

　　（六）实施民生工程，人民生活水平不断提高

　　居民收入稳步增长。积极拓展社会就业，安排促进就业和再就业资金5000万元，预计城镇新增就业11.8万人。市区企业退休人员养老金人均月增资124元，城镇老年居民养老补贴人均增加30元。预计，城市居民人均可支配收入、农民人均纯收入分别达23605元、11180元。社会保障不断完善。企业养老、基本医疗、失业等社会保险参保人数分别增加41.4万人、21.9万人、19.2万人。完善城镇居民医疗保险制度和职工医疗保险政策，扩大财政补助范围和标准。城市低保标准提高到月人均350元，农村低保标准低限提高到月人均220元，发放低保金1.5亿元。实行困难群众临时生活救助制度，向1.5万多户城镇低保户和社会困难户发放慈善物资，对6547名无业重残人员实施生活救助，为12575位90周岁以上高龄老人发放“尊老金”。生活条件持续改善。有效落实临时价格干预措施，全力保障粮油等生活必需品的市场供应和稳定。加强食品药品安全监管，全市食品药品检测合格率97.3%。完善住房保障体系，市区2708户家庭享受廉租住房保障，经济适用房竣工交付5700套。完成老新村整治及老住宅特修61.2万平方米，危旧房改造63万平方米。积极引导居民住房消费，房地产市场保持稳定。落实公交优先战略，简化市区公交票制，实行换乘优惠，34条线路降低票价，开通快速环路公交试验线，各类公交卡在市区162条公交线路上实现了全覆盖，初步形成城乡公交一体化发展格局。全年市级财政投入达到2.2亿元，公交高峰日运量突破百万人次，城市公共交通分担率提高2个百分点。

　　2008年是本届政府开局之年，市政府着力加强自身建设，全面履行各项职能，认真贯彻市人大及其常委会的各项决议，扎实办理人大代表的建议、批评、意见和政协委员的提案及建议案。大力加强民主法制建设，严格依法行政。认真贯彻《政府信息公开条例》，建立健全政府信息公开机制。做好第二次全国经济普查工作。强化应急体系建设，有效应对雪灾等突发公共事件。开展“五五”普法教育，推进平安无锡、法治无锡建设。畅通信访渠道，努力帮助群众排忧解难。加强安全生产监管，各类安全事故起数和死亡人数连续7年实现“双下降”。农机、审计、史志、档案、侨务、对台、人武、人防、双拥、民族宗教、气象、防震减灾、无线电管理等工作取得新成绩，妇女、儿童、老龄、残疾人等事业有了新发展。

　　去年，我国汶川等地遭遇了历史罕见的特大地震灾害。在中央和省统一部署下，我市圆满完成了抗震救灾各项任务。全市募集捐款3.7亿元，接收175名伤员来锡治疗，帮助1930名学生来锡就读，为灾区建成活动板房1400多套，汉旺、天池和清平等乡镇的灾后援建工作已全面展开，这充分体现了全市上下万众一心、和衷共济的强大凝聚力！

　　各位代表，过去一年，在迈向现代化的道路上，无锡取得了新的进步，常住人口人均地区生产总值突破1万美元，获得了“科学发展优秀城市”、“中国最具幸福感城市”等荣誉，城市影响力和美誉度进一步增强。这些成绩来之不易，凝聚着全市人民的智慧和辛劳。在此，我代表无锡市人民政府，向在各个领域和岗位上辛勤劳动、无私奉献的全体市民，向给予政府工作积极支持的人大代表和政协委员，向各民主党派、工商联、各人民团体和各界人士，向中央及省在锡单位，向驻锡人民解放军、武警官兵和公安干警，向所有参与、支持和关心无锡建设和发展的海内外朋友，表示崇高的敬意和由衷的感谢！

　　我们也清醒地看到，无锡的发展还面临许多亟须克服的困难和解决的矛盾：经济结构战略性调整任务依然艰巨，先进制造业和高端服务业发展需要进一步加快；城市现代化建设和城乡一体化发展面临许多新的课题，城市规划、建设和管理需要进一步加强；资源节约和环境保护是一项长期而艰巨的系统工程，节能减排工作需要进一步推进；社会事业发展相对滞后，政府公共服务能力需要进一步增强，等等。此外，政府工作中还存在一些不足和薄弱环节，政风建设还要不断加强。这些问题必须高度重视，在今后工作中切实加以解决。

2009年政府工作目标与总体要求

　　今年是建国六十周年，做好今年工作意义重大。当前，国内外经济环境发生了重大变化，我市经济增速放缓，部分企业经营困难，工业生产、外贸出口、消费需求的增长都呈回落态势，面临的不确定因素和困难增多。但应该看到，无锡已步入工业化后期和城市现代化建设的关键时期，消费升级和技术创新所激发的内部需求和内生动力依然旺盛，经济发展的基本态势没有逆转，仍有较好基础、较强活力和较大潜力。经过多年发展与调整，无锡具备较好的产业基础，有一大批具有较强核心竞争力的优势企业，经济结构也更趋合理，具备了抵御风险的能力。国内消费市场拓展空间广阔，投资增长和资本市场发展潜力巨大，中央和省对保增长、促转型采取了坚决有力的措施，我们要牢牢把握扩大内需政策带来的新机遇，努力构建新的竞争优势。抓住投资消费全面拉动的契机，积极发展商贸、物流、商务等服务业，促进现代服务业高端提升。抓住增值税转型、银根宽松和各种扶持政策聚焦的契机，推动企业加快技术改造、加强自主创新、淘汰落后产能，促进制造业转型升级。抓住国际大公司加速转移非核心业务的契机，大力引进服务外包产业、高新技术产业及其销售、研发中心，加快高端产业和人才集聚。抓住货币政策宽松、重大投资项目集中审批的契机，加快基础设施、生态环境、社会事业及新农村建设等一批重大项目建设，提升城市发展水平。总之，我们要正确认识所处的环境和形势，坚定发展信心，增强发展本领，落实发展举措，努力在应对挑战中把握机遇，在开拓进取中增创优势，继续走在改革发展的前列。

　　2009年政府工作的总体要求是：深入贯彻落实科学发展观，坚持以经济建设为中心，按照中央和省的决策部署，把保持经济稳定较快增长、推动结构调整和发展方式转变相结合，把扩大有效投资、积极拉动消费和改善民生相结合，深化改革开放增强经济社会发展活力和动力，加强社会建设加快解决涉及群众利益的热点难点问题，构筑无锡科学发展新优势，促进经济社会又好又快发展。

　　综合考虑各方面因素，2009年经济社会发展的主要预期目标是：地区生产总值增长11%；地方财政一般预算收入同口径增长11%；社会消费品零售总额增长16%；全社会固定资产投资增长20%；万元地区生产总值能耗降低4.5%，主要污染物排放量在2005年基础上累计削减16%，环境质量综合指数稳定达到85；城市居民人均可支配收入增长10%，农民人均纯收入增长10%；城镇登记失业率控制在3.8%以内；居民消费价格指数控制在省定范围以内。

　　实现发展目标，需要在“保增长、促转型、重统筹、惠民生”上下更大功夫。

　　——保增长，就是要全面优化经济增长动力结构。有效提高投资效率，加大政府性投资强度，大力支持企业发展，激发社会投资积极性。有效扩大消费需求，提升居民消费意愿和消费能力，增强消费需求对经济增长的拉动作用。有效调整出口产品结构，转变外贸增长方式，稳定出口形势。

　　——促转型，就是要全面推进城市现代化建设。坚持改革开放正确方向，着力完善科学发展的体制机制保障。坚持产业高端化发展导向，推动科技创新创业，提升企业自主创新能力和产业核心竞争力。坚持环保优先方针，进一步加大节能减排力度，推进生态文明建设，努力实现重点发展向优化发展的全面跨越。

　　——重统筹，就是要全面增强经济社会发展协调性。突出人口资源环境相协调，建设资源节约型、环境友好型城市。突出城乡相协调，构建城乡平等统一的制度框架和政策体系。突出改革发展稳定相协调，促进社会和谐，全面推进区域现代化进程。

　　——惠民生，就是要让群众共享改革发展成果。进一步落实积极的就业政策，千方百计增加城乡居民收入，安排好困难群众的生产生活。进一步加大财政保障力度，着力解决居住、出行、教育、医疗等涉及群众切身利益的现实问题，促进市民安居乐业。进一步完善社会保险、社会福利、社会救助体系，努力为市民提供均等化的公共服务、共享化的社会保障。

2009年政府工作主要任务

　　（一）更加注重扩大内需，全力以赴保增长促发展

　　坚持把扩大内需作为促进经济增长的着力点，全力落实中央、省、市扩大内需的各项措施，积极推动消费，努力扩大投资，实现又好又快发展。扩大消费需求。充分利用多年来我市城乡居民收入增长较快的有利条件，制订鼓励消费的政策，扩大消费市场，增强“以消费扩内需”的传导效应，培育新的消费热点。进一步改善政策环境与市场环境，大力鼓励居民自住型、改善型住房消费，保持房地产市场平稳健康发展。大力发展社区商业、农村商业、家政服务业，鼓励大型商贸连锁企业发展农村网点，新建放心连锁农家店150家以上。积极开发旅游、休闲、文化、健身等产业，促进消费结构加快升级。推动专业市场做大做强，抓好粮食科技物流中心等项目建设。加大投资力度。在优化结构的前提下扩大投资规模，发挥政府性投资引导作用，坚定企业投资信心，开工建设一批重大产业项目、基础设施和生态环境建设项目、民生工程项目。积极推进远景能源、开普动力、西格玛生化等先进制造业项目，全面开工建设火车站北广场综合交通枢纽、西水东中央生活区、崇安寺二期等城市综合体项目，加快太湖新城污水处理厂二期、锡东垃圾焚烧发电厂、500千伏江阴变等公用设施建设。做好对口援建四川灾区工作。提高服务水平。加强重点地区、重点行业、重点企业的运行监测，抓好生产要素组织，有效调节经济运行。完善土地供应政策，优先安排重大项目用地。取消和停止征收行政事业性收费140项，减轻企业负担。健全企业联系和服务制度，重点关注骨干企业和成长型中小企业，加大困难企业帮扶力度。鼓励金融机构加大信贷支持，完善金融服务，贷款规模增幅超全省平均水平。

　　（二）加快创新创业步伐，构建现代产业体系

　　统筹推进产业结构的调整和升级，提高自主创新能力，培育壮大特色优势产业，不断增强产业竞争力。完善产业规划。处理好新兴产业发展和传统产业改造的关系，科学制订现代产业体系总体规划和重点产业发展规划，引导传统优势产业加快提升，推动现代服务业、高新技术产业和先进制造业加快发展。推进产业集聚、企业集中，加快企业“退城进园”，努力形成资源配置合理、行业相对集中、分工合作有序的生产力布局。强化自主创新能力。积极开展科技创业促进年活动，发挥企业创新主体作用，完善创新环境，集聚创新资源，深入推进政产学研合作，促进科技成果加快转化为现实生产力。加快实施品牌与技术标准战略，建成“全国质量兴市先进市”。健全创新服务体系，设立专项资金，全力扶持重点研发机构、公共技术服务平台和企业技术中心建设，加快推进工业设计园等十大重点载体建设。加强创新人才队伍建设，继续实施“530”计划，积极发挥海内外科技创业领军人才作用。全社会研发费用占地区生产总值比重达到2.3%左右。突破重大产业项目。加快集成电路、新能源和生物医药等创新先导产业和新兴支柱产业发展，大力扶持软件和服务外包、现代物流、现代商贸和文化创意产业，建设一批农业标准化示范区，扶持一批竞争优势强、带动作用大的农业产业化项目，加快产业转型升级。高新技术产业增加值占规模以上工业增加值比重提高到43%以上。

　　（三）坚持改革开放基本方针，争创体制机制新优势

　　坚持社会主义市场经济的改革方向，全面推进《深化改革创新体制行动纲要》所确定的33项改革任务，激发经济社会发展的动力和活力。深化重点领域改革。继续优化国有经济布局和结构，推进国有企业改革发展，提高国有经济整体素质。发展农村三大合作，鼓励农村土地承包经营权以转包、出租、置换、入股等方式流转，帮助集体经济组织和农民增加收入。加快财税管理体制改革，加强预算和非税收入管理，优化财政支出结构。探索排污许可证有偿使用和交易制度，建立健全排污权交易平台和区域环境资源补偿机制。推进行政管理创新。完善社会事业管理体制，深化事业单位改革。建立健全市、市（县）区和乡镇（街道）三级行政服务体系，优化政务环境。创新城市管理模式，赋予乡镇（街道）及社区城市管理职能，将城市管理工作落实到基层。提升对外开放水平。加大利用外资力度，开展服务外包促进年活动，增创产业优势和发展特色。新增注册外资3000万美元以上重大项目30个，其中超亿美元项目3个以上；新增“123”计划服务外包企业20家。综合运用多种政策措施，稳定外贸发展形势，优化出口产品结构，巩固传统市场，开辟新兴市场。进出口贸易总额力争实现增长。积极引导境外投资，继续鼓励和支持企业“走出去”。加快开发区转型升级和优化发展，壮大产业集群，培育新的增长极。

　　（四）加大城乡建设力度，提升城市现代化水平

　　加快建设区域性中心城市，全面提升城乡基础设施承载能力和生态环境质量，推进城市现代化进程。加强基础设施和功能载体建设。加速太湖新城建设，基本建成骨架路网，完成一批公共设施项目建设；加快蠡湖新城“三集中”，完善蠡湖景区功能；全面启动锡东新城开发。推进京沪高速铁路无锡站和沪宁城际铁路无锡站、惠山站、新区站建设工程，开工建设城市快速轨道交通1号线。继续加大重点道桥建设力度，市区新增城市道路100公里以上，基本完成国省干线公路改造。进一步扩大口岸开放，完成无锡机场跑道改造和货运仓库建设，开拓国际航线3条左右。启动宜兴西氿、江阴第二水源等应急备用水源建设，确保城乡安全供水。狠抓环境综合整治。加强重点单位、重点领域节能减排工作，发展循环经济。推进管网全覆盖、污水全处理和垃圾机械化收集处理等环境工程建设。全面实施主要入湖河道及城区河道综合整治、引排通道建设等“太湖治理十大工程”，改善主要入湖河道水质，确保国控、省控断面水质稳定达标，实现主要污染物入湖总量下降、太湖富营养化程度下降。加快城乡生态建设。实施梅梁湖、竺山湖水域清淤，继续开展新一轮农村河道清淤。完成贡湖湾、尚贤河一期、长广溪二期等湿地建设，建成市区环太湖生态景观林带。严格保护耕地，大力推进“万顷良田”建设工程和宅基地置换整理。继续推进“绿色无锡”建设，全市造林4600公顷以上，市区新增公共绿地600万平方米以上，力争建成“国家森林城市”和“全国最佳人居环境城市”。

　　（五）建立健全制度安排，加快城乡一体化进程

　　统筹城乡发展，完善制度安排和政策体系，破除城乡二元结构，重点实施“十大强农惠农工程”，逐步实现社会保障和公共服务均等共享。推进城乡就业一体化。健全就业政策和就业管理服务体系，落实各项优惠扶持政策。加大就业补贴力度，对享受社会保险补贴的企业提高补贴标准，千方百计稳定和扩大社会就业。优化创业环境，加大创业培训和职业培训支持力度。构建和谐劳动关系，做好大学毕业生、下岗失业人员、城镇“零就业家庭”、农村低收入家庭等的就业援助帮扶工作。全年城镇新增就业8万人。推进城乡保障一体化。加快各层次社会养老保险制度的并轨和转换，逐步推进城镇居民医疗保险和新型农村合作医疗保险并轨。全市企业养老保险扩面10万人，基本医疗、失业、工伤等社会保险各扩面4万人。继续推进农民工、非公有制经济及城镇灵活就业人员、农村各类企业职工参加社会保险，农村养老保障综合覆盖率达到92%以上。继续提高企业退休人员基本养老金。完善最低生活保障制度，提高锡山区、惠山区农村低保水平，市区低保标准逐步实现城乡统一。健全社会救助体系，完善医疗救助结报制度。推进城乡公共服务一体化。加快社区事务工作站建设，农村社区服务中心建设率达70%，公共体育设施配建率达100%。建设一批示范性养老机构，养老福利床位新增1000张以上，居家养老机构实现城乡全覆盖。进一步推进市区城乡公交一体化，统一公交票制，调整优化公交线网，开通跨区域公交线路，延长营运里程及时间，加快公交智能化建设，切实提高公交运行、服务和监管水平。

　　（六）加强社会建设，改善居民生活质量

　　坚持以人为本，加快社会事业发展，全力办好26项为民办实事项目，不断提高居民生活水平。建设文明无锡。广泛开展群众性精神文明创建活动，加强公民道德建设，加强科普和法治教育，提升市民文明素质。全面推进历史街区和历史文化名镇、名村保护性修复，加快江阴、宜兴国家历史文化名城创建步伐。完善公共文化设施，加快建设无锡大剧院、报业大厦等工程，建成功能完备的盲人图书馆。培育具有活力和竞争力的文化发展主体。全力办好第二届世界佛教论坛和市第十届运动会。建设教育强市。构建全民教育体系，加快建设全民学习、终身学习的学习型社会。完善义务教育投入保障机制，发展学前教育，关心特殊教育，推动高等教育和职业教育水平提升。加快教育资源整合和布局调整，建成青山高中、辅仁高中、公益职校新校区，完成聋哑学校改造搬迁。鼓励和规范民办教育发展，深化教育国际交流合作。建设健康城市。坚持公共医疗卫生公益性质，加大财政保障力度，完善社区卫生服务网络，健全公共卫生服务体系。深化公立医院改革，提升医疗技术服务水平，鼓励社会力量依法兴办医疗服务机构。加快市中医院、二院、三院、妇幼保健院等新改扩建。推进人口和计划生育服务体系建设，提高出生人口素质，完善人口服务管理机制。健全全民健身服务体系，实施体育基本现代化试点工作。建设和谐无锡。加快保障性安居工程建设，为2800户以上家庭提供廉租住房保障，解决家庭住房面积16平方米以下低保家庭的住房困难。加快城区危旧房和城中村改造，完成规模化老新村整治80万平方米。强化食品药品监管，市区建立肉食品屠宰、批发和销售全过程监管体系，保障群众生活安全。坚持安全发展，落实企业安全生产主体责任。加强应急管理，健全社会预警机制、突发公共事件应急机制和社会动员机制。加强国防后备力量建设，做好双拥工作。积极防范、妥善处置各类不稳定因素，有效化解社会矛盾。完善大调解机制，健全大防控体系，努力保持社会安定有序，增进社会和谐。

　　（七）提高行政效能，建设服务型政府

　　保持奋发有为的精神状态，以转变政府职能为核心，以提高行政效能为重点，全面加强政府自身建设。严格依法行政。依照法定权限和程序行使权力、履行职责，依法管理经济社会事务。自觉接受人大法律监督、工作监督和政协民主监督，坚持重大事项向人大报告和向政协通报制度。落实行政执法责任制，加强行政复议、行政监察和执法监督，规范政府行政行为。不断完善社情民意反应机制，推进政务公开，健全政府信息发布制度，提高政府工作透明度。强化制度保障。完善重大事项专家咨询、社会听证、社会公示制度，进一步构建科学民主决策机制。健全政府工作绩效评估制度，科学制订评价和考核体系。强化行政问责制，狠抓任务落实，切实增强政府执行力。加强作风建设。继续推进机关效能建设，提高工作效率和服务质量，降低行政成本。深化学习实践科学发展观试点活动，加强公务员教育、培训、管理，努力建设一支政治坚定、业务精湛、作风优良、清正廉洁的高素质公务员队伍。

　　各位代表，无锡现代化建设的任务繁重而艰巨，前景美好而光明！让我们紧密团结在以胡锦涛同志为总书记的党中央周围，在中共无锡市委领导下，全面落实科学发展观，聚精会神搞建设，一心一意谋发展，为率先基本实现现代化而努力奋斗！
